# Shah-Waardenburg Syndrome

**DOI:** 10.11604/pamj.2013.14.60.1543

**Published:** 2013-02-12

**Authors:** Abdelhalim Mahmoudi, Mohamed Rami, Khalid Khattala, Aziz Elmadi, My Abderrahmane Afifi, Bouabdallah Youssef

**Affiliations:** 1 Department of Pediatric Surgery, University Hospital Hassan II, Fez, Morocco

**Keywords:** Intestinal aganglionosis, Hirschsprung's disease, Waardenburg-Shah syndrome, neurocristopathy

## Abstract

Shah-Waardenburg syndrome (SWS) is a neurocristopathy and is characterized by Hirschsprung's disease (HD), deafness, and depigmentation of hairs, skin, and iris. Is a very rare congenital disorder with variable clinical expression. This report describes a 4-day-old male newborn with Waardenburg's syndrome associated with aganglionosis of the colon and terminal ileum, and review the relevant literature for draws attention to the causal relationship between these two entities.

## Introduction

Waardenburg syndrome (WS) is an autosomal recessive neurocristopathy with variable presentation. Four types of WS are described. Type-IV is the association of WS with Hirschsprung&apos;s disease (HD). This type is called Shah- Waardenburg syndrome (SWS). The classic presentations of SWS include HD, sensorineural deafness, and depigmentation of hairs, skin, and iris [1]. In patients with SWS, the aganglionic segment may be long and may have total colonic or total intestinal aganglionosis [2, 3]. The authors present such a case of long segment aganglionosis in a 4-day-old male with Waardenburg-Shah syndrome and discuss diagnosis, treatment, and prognosis.

## Patient and observation

**Figure 1:**
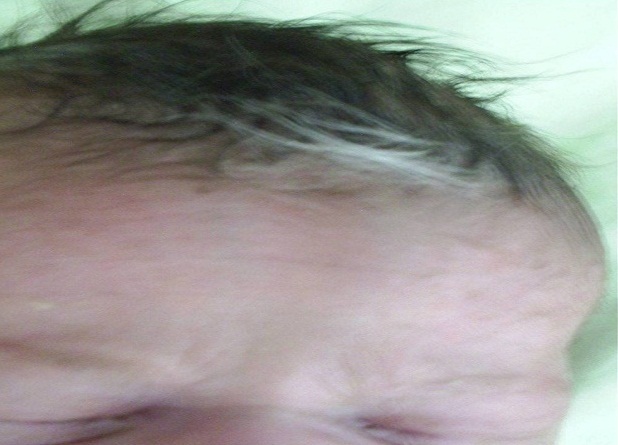
The patient had had a white forelock of hair

**Figure 2:**
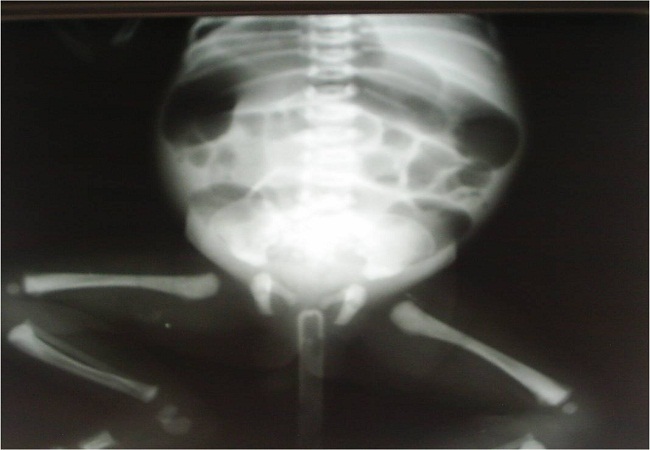
Radiographic X-Ray revealed dilated bowel loops but no air-fluid levels or pelvic gas

**Figure 3:**
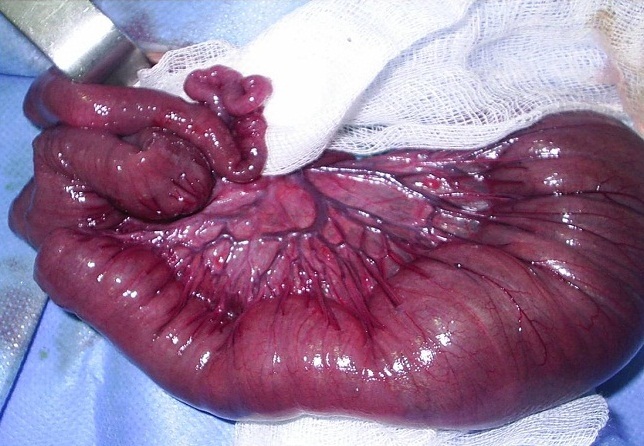
Intra-operatively, the colon and distal ileum were found contracted; the ileum proximal to the transition zone was distended

A 4-day-old full term male baby with a birthweight of 3100 g was admitted with history of bilious vomiting, inability to pass meconium, and abdominal distension since birth. On examination, he had a white forelock of hair and massively distended abdomen, pale irises, and absence of reaction to any sound (Figure 1). Radiographic investigations revealed dilated bowel loops but no air-fluid levels or pelvic gas (Figure 2). The barium enema was normal. The patient was given intravenous fluid resuscitation and nasogastric decompression and operated on the fifth day of life. An exploratory laparotomy was undertaken revealed distended proximal jejunal and ileal loops, the 30 cm of terminal ileum and the colon were contracted (Figure 3). Multiple sero-muscular biopsies were taken from colon and terminal ileum; appendectomy was also performed. A divided ileostomy was performed at the transition zone. Histological examination of gut biopsies showed aganglionosis in colon and terminal ileum, compatible with Hirschsprung disease. A diagnosis of Shah- Waardenburg syndrome was made. The enterostomies started to function on the second postoperative day, and he started to gain weight. However, the baby died because of sepsis at 4 weeks of age.

## Discussion

WS4 is the association of Waardenburg syndrome with Hirschsprung disease. Only 48cases are reported in English literature till 2002 [4]. The coexistence of WS and HD is quite rare. The incidence of WS is 2/100,000 and of HD 2/10,000. The probability of the 2 conditions existing together is therefore 4 in 1 million according to the known number of cases. The incidence of congenital deafness is 1/1000 and coincidental concurrence with HD would be expected at a rate of 1 in 5 million [5]. Shah-Waardenburg syndrome (SWS) is an autosomal recessive condition. Several gene alterations have been described to play a distinctive role in the development of SWS such as endothelin (EDN), EDNRB, and SOX10 genes [6]. A SOX10 mutation is now considered an important cause of neurocristopathies including SWS [7]. Other gene mutation such as EDN3 and EDN B receptor EDNRB (chromosome 13) may support the idea that HD might be a result of disturbed EDN signaling pathways. The clinical findings of the patient and the age at presentation can vary according to the length of the involved segment. Patients with short segment involvement mostly present at a more advanced age with chronic constipation, malabsorption, and enterocolitis, whereas those with long segment involvement present with intestinal obstruction findings such as bilious vomiting, abdominal distension, and inability to feed orally from the first few days of life [5, 8–10]. There are five major and five minor diagnostic criteria for Waardenburg syndrome. Major criteria include sensorineural hearing loss, iris pigmentary abnormality (two eyes different color or irisbicolor or characteristic brilliant blue iris), hair hypopigmentation (white forelock or white hairs at other sites on the body), dystopia canthorum (lateral displacement of inner canthi) and first-degree relative previously diagnosed with Waardenburg syndrome. Minor criteria include skin hypopigmentation (congenital leukoderma/ white skin patches), medial eyebrow flare (synophrys), broad nasal root, hypoplasia alae nasi, and premature graying of the hair.

The clinical diagnosis of WS requires at least 2 major or one major and one minor criteria [11, 12]. The diagnosis of HD in type IV WS patients is made using the history, physical examination, plain abdominal x-ray, barium enema, anorectal manometry, and rectal biopsy. A barium enema is frequently not diagnostic in young infants with extensive aganglionosis, necessitating a laparotomy for small intestinal biopsy specimens to identify the level of aganglionosis [13]. The full-thickness intestinal biopsy specimens taken during surgery and investigation of the appendix specimen enabled a definite diagnosis and determination of the aganglionic segment length.

The length of the aganglionic segment involved affects the clinical course of the disease and is also very important in surgical treatment planning. Most of the previous series used various procedures after the initial enterostomy such as the Soave endorectal pull-through for short segments, the modified extended Duhamel method for long segments, the Swenson pull-through, and the Kimura-Stringel operation [3, 14]. Postoperative complications in type IV WS patients with EA characteristics are no different than those seen in short bowel syndromes. These cases are faced with fluid electrolyte imbalance, bacterial overgrowth, and TPN and catheter-related complications (sepsis, catheter blockage, liver dysfunction) at the early stage. The mortality in these patients is directly related to sepsis and hepatic failure [15, 16]. Our patients also died in the first month because of sepsis.

## Conclusion

Shah-Waardenburg syndrome is a very rare syndrome with a higher incidence of Total colonic aganglionosis with or without small bowel involvement which leads to high morbidity and mortality in the neonatal age group.
